# Supplementary material for: Low chorionic villous succinate accumulation associates with recurrent spontaneous abortion risk
Source: Nat Commun. 2021 Jun 8;12:3428. doi: 10.1038/s41467-021-23827-0 (PMC8187647; doi:10.1038/s41467-021-23827-0)
Supplement: Supplementary file 1 — Supplementary Information [file 41467_2021_23827_MOESM1_ESM.pdf]

Supplementary Information

Low embryonic villous succinate accumulation associates with recurrent spontaneous abortion risk

Wang et al

Supplementary Figure 1

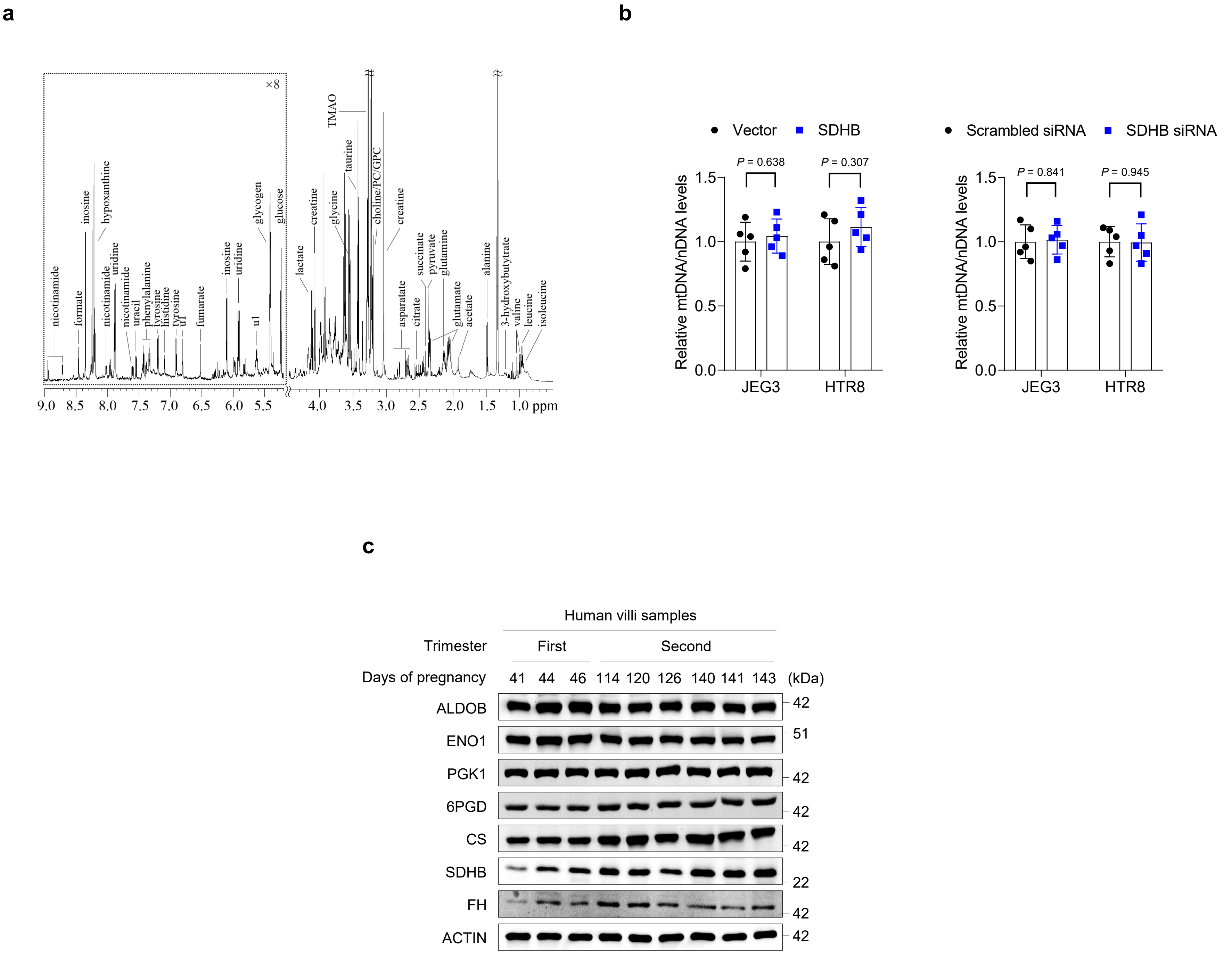

Supplementary Figure 1. Metabolic profile in human villi samples. a the two-dimensional (2D) NMR spectra of metabolites detected in human tissue samples. The NMR raw data are deposited in the BMRbig database (https://bmrbig.org/released/bmrbig7) with identifier "bmrbig7". **b** Mitochondrial levels in cells with various treatments (n = 5 independent experiments). **c** Protein expression levels in villi samples from normal pregnant. **b** Data represent the mean ± standard error. Two-tailed unpaired Student's t-tests in **b**. Source data are provided as a Source Data file.

Supplementary Figure 2

a

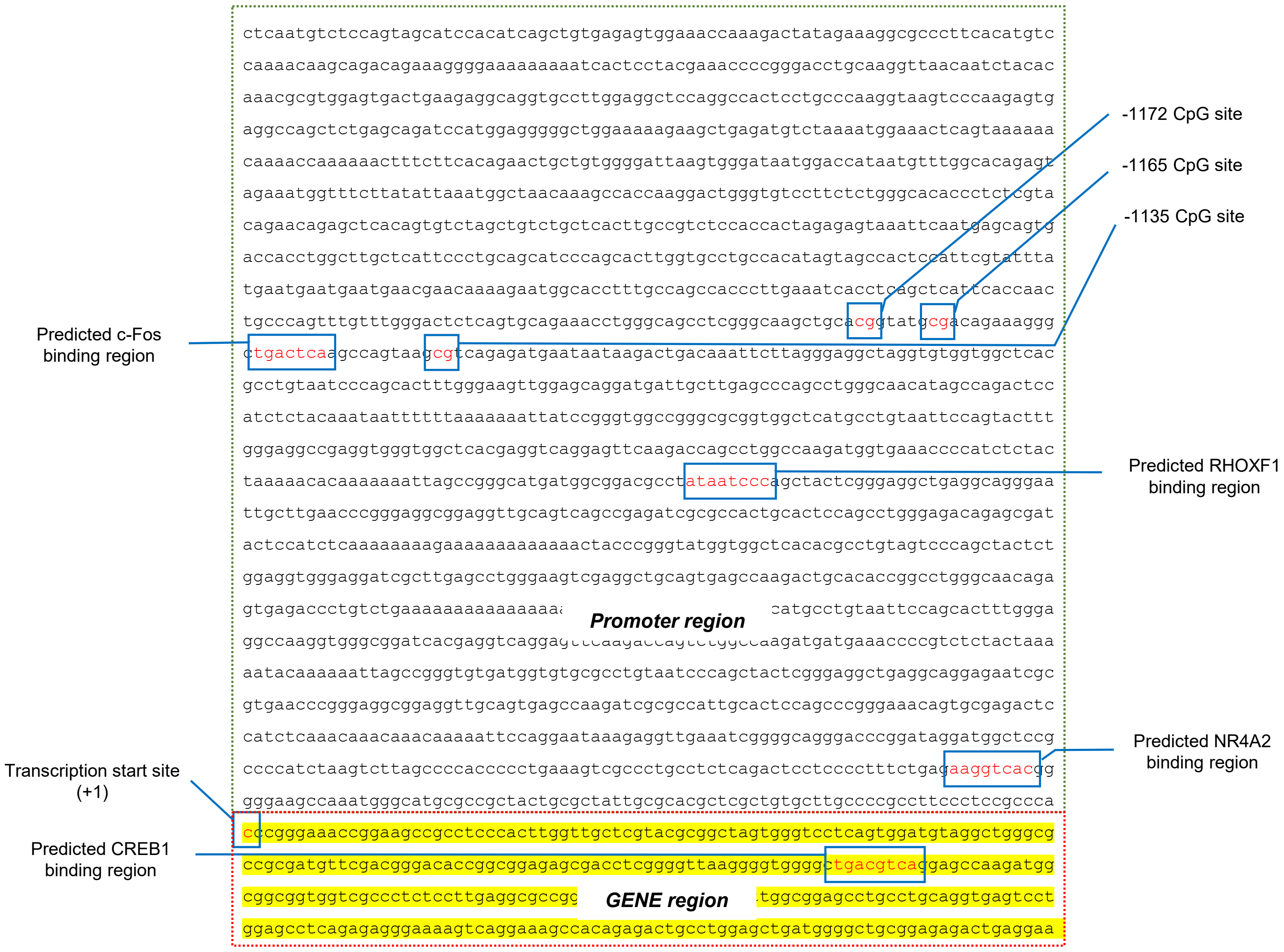

Transcriptional factor	Score	Predicted site sequence
CREB1	1.00	tgacgtca
c-Fos	1.00	tgactca
NR4A2	0.99	aaggtcac
RHOXF1	0.99	ataatccc

b

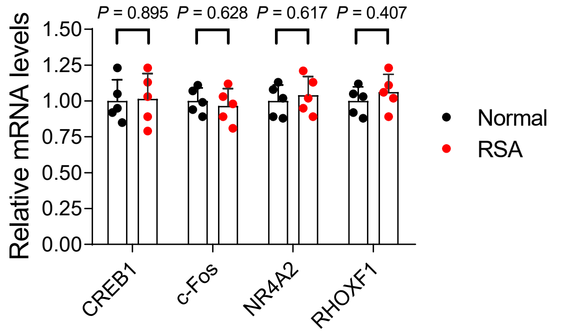

c

d

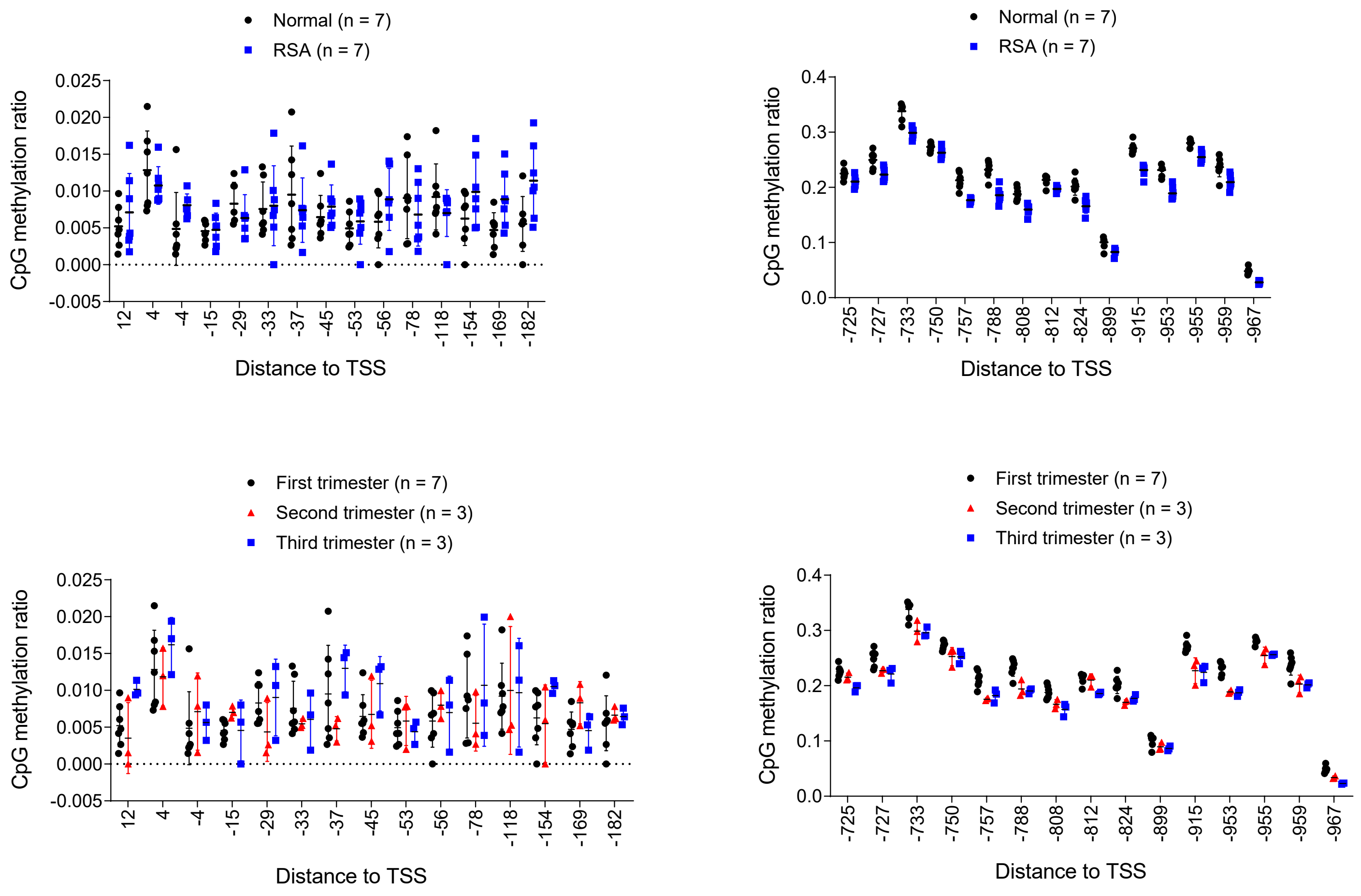

Supplementary Figure 2. Annotation of SDHB promoter region. a The -1172/-1165/-1135 CpG sites, and the predicted transcriptional factor binding sites, were indicated in the SDHB promoter. **b** The expression of transcriptional factors in villi samples from RSA patients (n = 5 biologically independent samples) and normal controls (n = 5 biologically independent samples). **c** Methylation levels of SDHB promoters in human villi samples. **b-d** Data represent the mean ± standard error. Two-tailed unpaired Student's t-tests in **b**. Source data are provided as a Source Data file.

Supplementary Figure 3

a

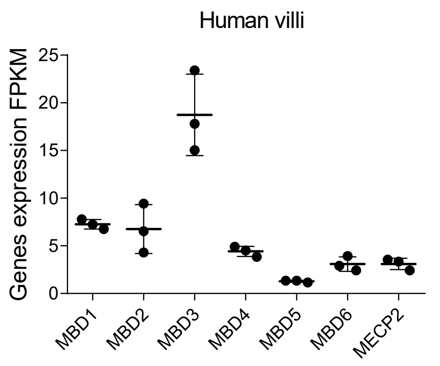

b

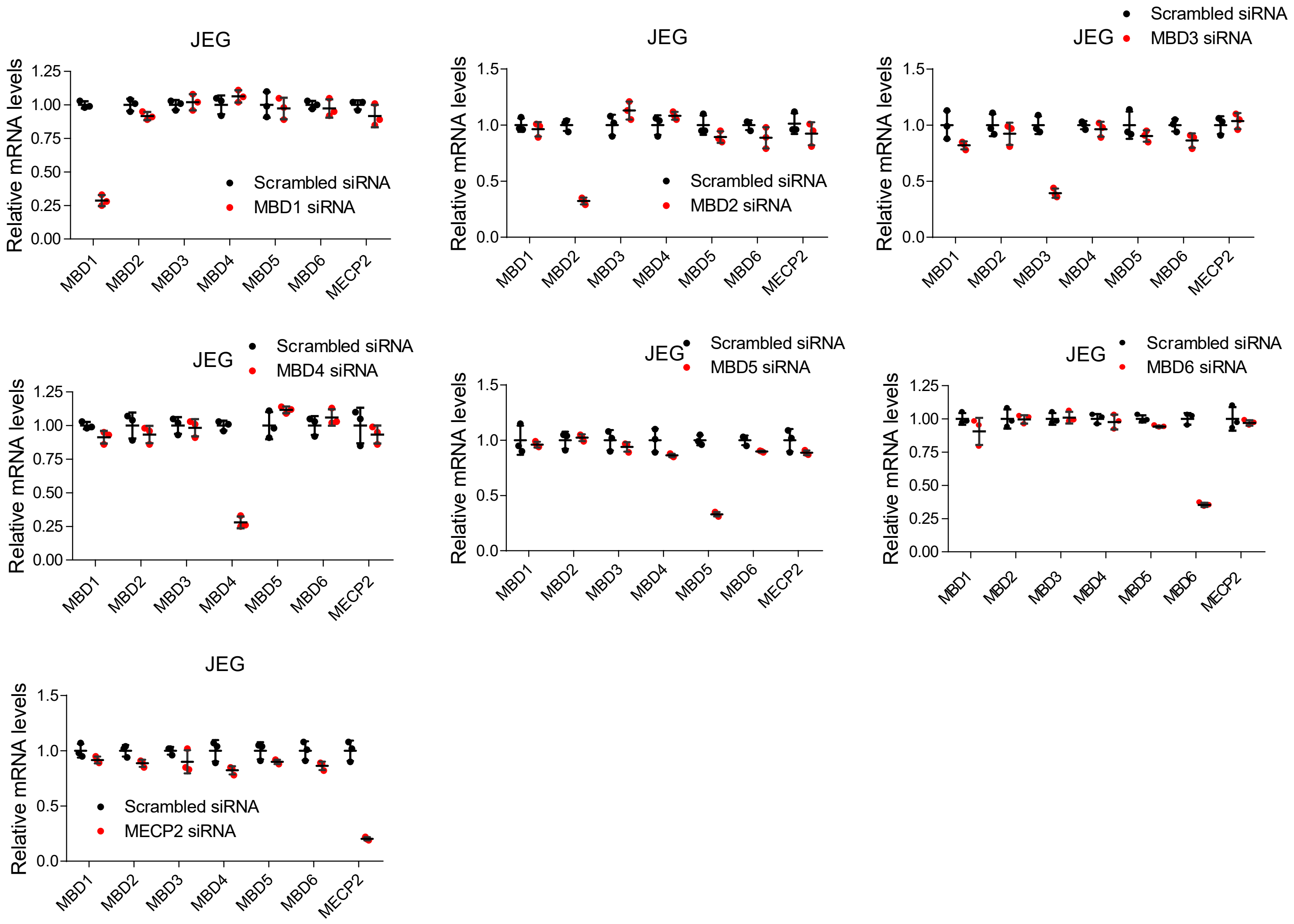

c

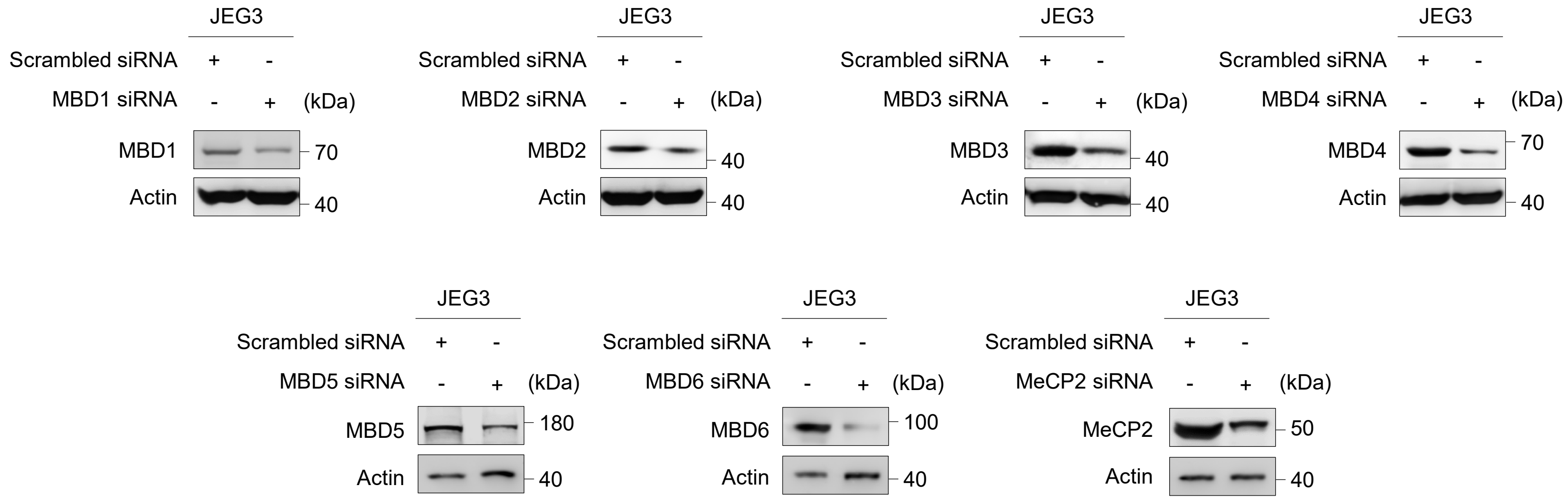

Supplementary Figure 3. The mRNA levels of methyl-CpG-binding proteins in villi from human normal pregnant (**a**) (n = 3 biologically independent samples) and the knockdown efficiency of each methyl-CpG-binding protein in cultured JEG3 cells (**b**, **c**) (**b**, n = 3 independent experiments). **a**, **b** Data represent the mean ± standard error. Source data are provided as a Source Data file.

Supplementary Figure 4

a

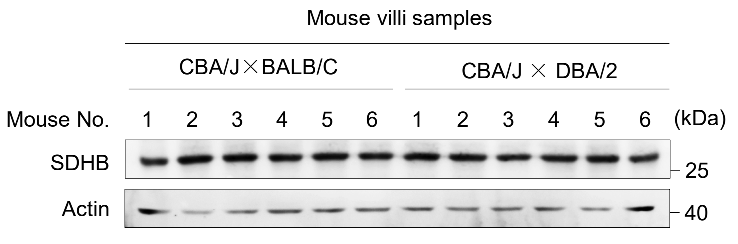

b

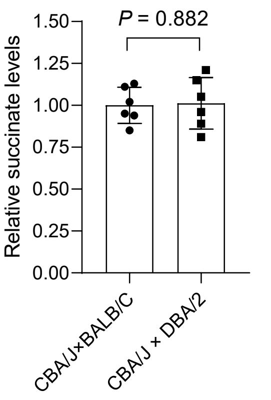

Supplementary Figure 4. The SDHB protein levels (**a**) and succinate levels (**b**) in villi (n = 6 biologically independent samples) from either CBA/J×BALB/C or CBA/J × DBA/2 mouse models (n = 5 biologically independent samples). **b** Data represent the mean ± standard error. ns not significant. Two-tailed unpaired Student's t-tests in **b**. Source data are provided as a Source Data file.

Supplementary Figure 5

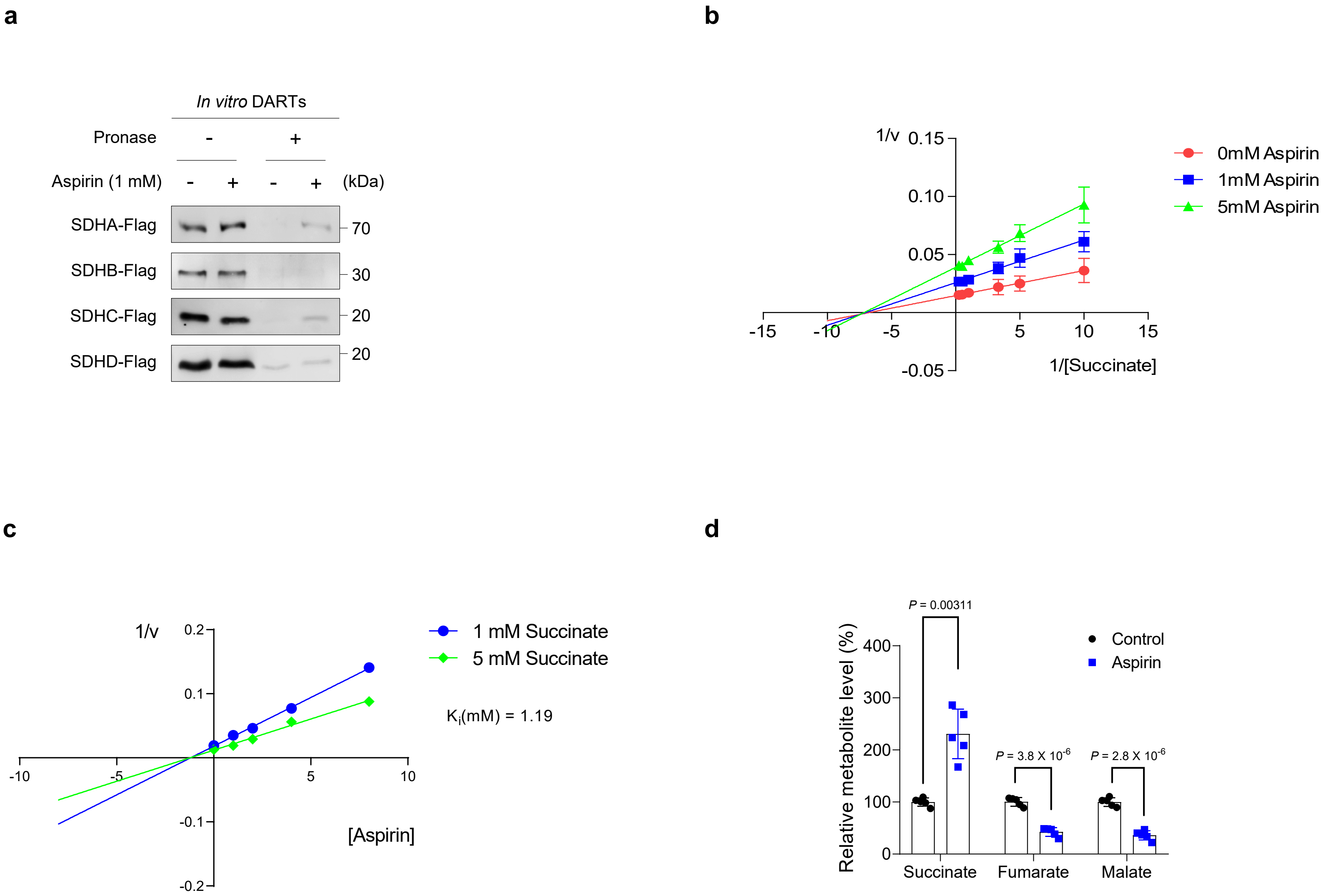

Supplementary Figure 5. Aspirin accumulates succinate by inhibiting SDH. a DARTS with aspirin confirms aspirin interacts with SDH components SDHA and SDHC. **b** Lineweaver-Burk plots showed that Aspirin uncompetitively inhibited SDH (n = 3 independent experiments). **c** The inhibitory constant (Ki) was calculated from a Dixon plot. **d** Relative levels of intracellular succinate, fumarate, and malate in JEG3 cells treated with or without 1 mM Aspirin. **b**, **d** Data represent the mean ± standard error. Two-tailed unpaired Student's t-tests in **d**. Source data are provided as a Source Data file.

Supplementary Table 1. Metabolite concentrations in decidua from individuals with RSA or normal controls

Metabolites (μmol/g)	Normal (n = 29)	RSA (n = 29)	*P* value	Adjusted *P* value
Glucose	3.286 ± 0.965	3.071 ± 0.669	0.330	0.866
Pyruvate	0.279 ± 0.093	0.298 ± 0.089	0.437	0.866
Lactate	3.004 ± 1.158	2.932 ± 0.958	0.797	0.874
Citrate	0.263 ± 0.076	0.279 ± 0.071	0.426	0.866
Succinate	0.465 ± 0.111	0.501 ± 0.108	0.221	0.866
Fumarate	0.159 ± 0.06	0.166 ± 0.06	0.654	0.874
Phenylalanine	0.198 ± 0.053	0.207 ± 0.05	0.514	0.866
Tyrosine	0.189 ± 0.055	0.198 ± 0.055	0.524	0.866
Asparatate	1.404 ± 0.454	1.559 ± 0.404	0.177	0.866
Glutamate	1.908 ± 0.68	2.042 ± 0.672	0.455	0.866
Glutamine	2.654 ± 0.833	2.769 ± 0.727	0.579	0.866
Glycine	1.488 ± 0.564	1.538 ± 0.611	0.746	0.874
Valine	0.447 ± 0.099	0.46 ± 0.09	0.599	0.866
Isoleucine	0.251 ± 0.069	0.257 ± 0.06	0.730	0.874
Leucine	0.464 ± 0.13	0.481 ± 0.122	0.603	0.866
Histidine	0.136 ± 0.049	0.146 ± 0.04	0.374	0.866
Alanine	1.128 ± 0.268	1.116 ± 0.242	0.851	0.876
Acetate	0.281 ± 0.059	0.289 ± 0.067	0.610	0.866
Formate	0.419 ± 0.17	0.35 ± 0.145	0.106	0.866
Inosine	0.318 ± 0.151	0.338 ± 0.162	0.618	0.866
Creatine	1.019 ± 0.494	1.176 ± 0.647	0.302	0.866
Hypoxanthine	0.672 ± 0.283	0.721 ± 0.27	0.497	0.866
Carnosine	0.019 ± 0.012	0.019 ± 0.01	0.910	0.910
Uridine	0.37 ± 0.19	0.399 ± 0.188	0.564	0.866
Taurine	4.089 ± 1.612	4.207 ± 1.689	0.787	0.874
3-hydroxybutytrate	0.308 ± 0.111	0.334 ± 0.154	0.459	0.866
Nicotinamide	0.126 ± 0.066	0.129 ± 0.065	0.839	0.876
NAD	0.016 ± 0.007	0.017 ± 0.009	0.409	0.866
AMP	0.009 ± 0.005	0.007 ± 0.004	0.420	0.866
ADP	0.016 ± 0.01	0.017 ± 0.009	0.756	0.874
Uracil	0.211 ± 0.066	0.228 ± 0.059	0.297	0.866

Data presented are given in μmol/g (mean ± SD). *P* values were derived from unpaired two-sample *t* test (two groups have the same SD) or unpaired two-sample *t* test with Welch's correction (two groups do not have the equal SD) and then corrected by using the Benjamini & Hochberg method for multiple sampling (adjusted *P* value). Source data are provided as a Source Data file. NAD, Nicotinamide adenine dinucleotide; AMP, Adenosine monophosphate; ADP, Adenosine diphosphate.

Supplementary Table 2. Genotyping of *SDHB* promoter region SNPs identified in normal pregnant women and RSA patients

SNP IN dbSNP	Position(base on db cDNA(NM_003000.3))	Frequency in Normal Group			Frequency in RSA Group		
		wild-type homozygosity	heterozygosity	mutant homozygosity	wild-type homozygosity	heterozygosity	mutant homozygosity
rs2647209	c.-2048A>G	9/10	0/10	1/10	19/20	0/20	1/20
rs3754509	c.-1986A>G	4/10	1/10	5/10	10/20	3/20	7/20
rs202157870	c.-1967delA	10/10	0/10	0/10	19/20	0/20	1/20
rs2746461	c.-1425C>T	9/10	0/10	1/10	19/20	0/20	1/20
rs988711595	c.-1028C>T	10/10	0/10	0/10	19/20	0/20	1/20
rs370466545	c.-888A>G	10/10	0/10	0/10	19/20	0/20	1/20
rs148969634	c.-561G>A	10/10	0/10	0/10	19/20	0/20	1/20
rs2647211	c.-444C>T	9/10	0/10	1/10	19/20	0/20	1/20
NEW	c.-216T>C	10/10	0/10	0/10	19/20	0/20	1/20

The number of sequenced normal pregnant women is 10 and the number of RSA patients is 20. Frequency of homozygous or heterozygous SNPs is listed in terms of fractions.

Supplementary Table 3. Primer sequence information

Name	Sequence (5'-3')	Purpose
SDHB-forward (Xho I)	CCCTCGAGATGGCGGCGGTGGTCGCACTCTCCT	Clone
SDHB-reverse (EcoR I)	CGGAATTCAACTGAAGCTTTCTTCTCCTTATAG	Clone
siMBD1(homo)	GCTGTGAGAACTGTGGAAT	siRNA Targets
siMBD2(homo)	GCAAGGTACCTGGGAAATA	siRNA Targets
siMBD3(homo)	GCAGCTCCTCTGCAAACAA	siRNA Targets
siMBD4(homo)	GCAAAGAAGATGTTGCTAT	siRNA Targets
siMBD5(homo)	GCTTGGAATGTCCTCTTAT	siRNA Targets
siMBD6(homo)	GCACAGAGCTGTCTTCCTT	siRNA Targets
siMECP2(homo)	AGAGAAAGAGGGCAAGCAT	siRNA Targets
SDHB-KD	CCGATTTGAGCAACTTCTATG	shRNA Targets
VHL-KD	GATCTGGAAGACCACCCAAAT	shRNA Targets
PHD2-KD	CTGTTATCTAGCTGAGTTCAT	shRNA Targets
ACTB-Forward	TCCCTGGAGAAGAGCTACG	qRT-PCR
ACTB-Reverse	GTAGTTTCGTGGATGCCACA	qRT-PCR
HK1--Forward	GCTCTCCGATGAAACTCTCATAG	qRT-PCR
HK1--Reverse	GGACCTTACGAATGTTGGCAA	qRT-PCR
GPI--Forward	CAAGGACCGCTTCAACCACTT	qRT-PCR
GPI--Reverse	CCAGGATGGGTGTGTTTGACC	qRT-PCR
TPI-Forward	CTCATCGGCACTCTGAACG	qRT-PCR
TPI-Reverse	GCGAAGTCGATATAGGCAGTAGG	qRT-PCR
PGK1-Forward	TGGACGTTAAAGGGAAGCGG	qRT-PCR
PGK1-Reverse	GCTCATAAGGACTACCGACTTGG	qRT-PCR
ENO1-Forward	AAAGCTGGTGCCGTTGAGAA	qRT-PCR
ENO1-Reverse	GGTTGTGGTAAACCTCTGCTC	qRT-PCR
PKM2-Forward	ATGTCGAAGCCCCATAGTGAA	qRT-PCR
PKM2-Reverse	TGGGTGGTGAATCAATGTCCA	qRT-PCR
6PGD-Forward	ATGGCCCAAGCTGACATCG	qRT-PCR
6PGD-Reverse	AAAGCCGTGGTCATTCATGTT	qRT-PCR
TKT-Forward	TCCACACCATGCGCTACAAG	qRT-PCR
TKT-Reverse	CAAGTCGGAGCTGATCTTCCT	qRT-PCR
PDHA1-Forward	TGGTAGCATCCCGTAATTTTGC	qRT-PCR
PDHA1-Reverse	ATTCGGCGTACAGTCTGCATC	qRT-PCR
CS-Forward	TGCTTCCTCCACGAATTTGAAA	qRT-PCR
CS-Reverse	CCACCATACATCATGTCCACAG	qRT-PCR
IDH1-Forward	TGTGGTAGAGATGCAAGGAGA	qRT-PCR
IDH1-Reverse	TTGGTGACTTGGTCGTTGGTG	qRT-PCR
IDH2-Forward	CGCCACTATGCCGACAAAAG	qRT-PCR
IDH2-Reverse	ACTGCCAGATAATACGGGTCA	qRT-PCR
IDH3A-Forward	CCCGCGTGGATCTCTAAGG	qRT-PCR
IDH3A-Reverse	AATTTCTGGGCCAATACCATCTC	qRT-PCR
OGDH-Forward	GGCTTCCCAGACTGTTAAGAC	qRT-PCR

OGDH-Reverse	GCAGAATAGCACCGAATCTGTTG	qRT-PCR
SUCLA2-Forward	TCTCCGTTCCCAAAGGATATGT	qRT-PCR
SUCLA2-Reverse	CACCAGCTAAAACCTGTGCC	qRT-PCR
SDHA-Forward	CAAACAGGAACCCGAGGTTTT	qRT-PCR
SDHA-Reverse	CAGCTTGGTAACACATGCTGTAT	qRT-PCR
SDHB-Forward	ACAGCTCCCCGTATCAAGAAA	qRT-PCR
SDHB-Reverse	GCATGATCTTCGGAAGGTCAA	qRT-PCR
SDHC-Forward	CTGTTGCTGAGACACGTTGGT	qRT-PCR
SDHC-Reverse	ACAGAGGACGGTTTGAACCTA	qRT-PCR
FH-Forward	GGAGGTGTGACAGAACGCAT	qRT-PCR
FH-Reverse	CATCTGCTGCCTTCATTATTGC	qRT-PCR
MDH2-Forward	TCGGCCCAGAACAATGCTAAA	qRT-PCR
MDH2-Reverse	GCGGCTTTGGTCTCGATGT	qRT-PCR
SDHB Promoter Region(-1165/-1172 CpG)(Non-methylated)	GGCAAGCTGCACGGTATGCGACAGAAAGGG	EMSA
SDHB Promoter Region(-1165/-1172 CpG)(Methylated)	GGCAAGCTGCAC(Me)GGTATGC(Me)GACAGAAAGGG	EMSA
SDHB Promoter Region(-1135 CpG)(Non-methylated)	ACTCAAGCCAGTAAGCGTCAGAGATGAATA	EMSA
SDHB Promoter Region(-1135 CpG)(Methylated)	ACTCAAGCCAGTAAGC(Me)GTCAGAGATGAATA	EMSA
SDHB Promoter c-Fos Binding Region	ACAGAAAGGGCTGACTCAAGCCAGTAAGCG	EMSA
SDHB Promoter Region(-1165/-1172 CpG)(Non-methylated)	GGCAAGCTGCACGGTATGCGACAGAAAGGG	EMSA
SDHB-methyl-1F	GAGTAATTAAGTGGGAGGyGGTTTT	methylation
SDHB-methyl-1R	AACTCCATCTCAAACAAACAAACA	methylation
SDHB-methyl-2F	TTGTTTTTTAGGTTGGAGTGTAGTG	methylation
SDHB-methyl-2R	CAAAAATTCAAAACCAACCTAACC	methylation
SDHB-methyl-3F	GGTTAGGTTGGTTTTGAATTTTTG	methylation
SDHB-methyl-3R	ACTTAAACCCAACCTAAACAACATAA	methylation
SDHB-methyl-4F	TTATGTTGTTTAGGTTGGGTTTAAGT	methylation
SDHB-methyl-4R	CCCTTAAAATCACCTCAACTCATTC	methylation
SDHB-methyl-5F	GAATGAGTTGAGGTGATTTTAAGGG	methylation
SDHB-methyl-5R	ACCAAAAACTAAATATCCTTCTCTAAACA	methylation
SDHB-Region1-F	CGCCGCTACTGCGCTATT	ChIP-PCR
SDHB-Region1-R	CCTTGCCCTATGCTTCCT	ChIP-PCR
SDHB-Region2-F	ACAGTGCGAGACTCCATC	ChIP-PCR

SDHB-Region2-R	CGTCAGCCCCACCCCTTA	ChIP-PCR
SDHB-Region3-F	CAGTGGCTCATGCCTGTAAT	ChIP-PCR
SDHB-Region3-R	GGGGTGGGGCTAAGACTTAGA	ChIP-PCR
SDHB-Region4-F	GGCGGACGCCTATAATCC	ChIP-PCR
SDHB-Region4-R	CCACCTTGGCCTCCCAAAGT	ChIP-PCR
SDHB-Region5-F	GGAAGTTGGAGCAGGATG	ChIP-PCR
SDHB-Region5-R	GATGGAGTATCGCTCTGTCTC	ChIP-PCR
SDHB-Region6-F	CTTTGCCAGCCACCCTTGA	ChIP-PCR
SDHB-Region6-R	ACCTCGTGAGCCACCCACCT	ChIP-PCR
SDHB-Region7-F	GGGATTAAGTGGGATAATGGA	ChIP-PCR
SDHB-Region7-R	ACTGAGAGTCCCAAACAAACT	ChIP-PCR
16S rRNA (mtDNA)-F	GCCTTCCCCCGTAAATGATA	Mitochondrial Mass Quantification
16S rRNA (mtDNA)-R	TTATGCGATTACCGGGCTCT	Mitochondrial Mass Quantification
β2-microglobulin (nDNA)-F	TGCTGTCTCCATGTTTGATGTAT	Mitochondrial Mass Quantification
β2-microglobulin (nDNA)-R	TCTCTGCTCCCCACCTCTAAGT	Mitochondrial Mass Quantification
SDHB-1F	CACAAGACAGCCACGTGAATC	Sanger Sequencing
SDHB-1R	CCAGTCCTTGGTGGCTTTGT	Sanger Sequencing
SDHB-2F	GGGCTGGAAAAAGAAGCTGAGAT	Sanger Sequencing
SDHB-2R	CCTAGCCTCCCTAAGAATTTGTCAGT	Sanger Sequencing
SDHB-3F	CACCCTTGAAATCACCTCAGCTC	Sanger Sequencing
SDHB-3R1	TTCTCAGAAAGGGGAGGAGTCTGA	Sanger Sequencing
SDHB-3R	GCAGTCTCTGTGGCTTTCCTGACTTT	Sanger Sequencing
SDHB-3SEQF	GGTATGGTGGCTCACACG	Sanger Sequencing
SDHB-3SEQR	TTTTCAGACAGGGTCTCACTCT	Sanger Sequencing

Supplementary Table 4. STROBE Statement—Checklist of items that should be included in reports of *case-control studies*

	Item No	Recommendation	Page No
**Title and abstract**	1	(*a*) Indicate the study's design with a commonly used term in the title or the abstract	2
		(*b*) Provide in the abstract an informative and balanced summary of what was done and what was found	2
**Introduction**			
Background/rationale	2	Explain the scientific background and rationale for the investigation being reported	3-5
Objectives	3	State specific objectives, including any prespecified hypotheses	5
**Methods**			
Study design	4	Present key elements of study design early in the paper	16-19
Setting	5	Describe the setting, locations, and relevant dates, including periods of recruitment, exposure, follow-up, and data collection	16
Participants	6	(*a*) Give the eligibility criteria, and the sources and methods of case ascertainment and control selection. Give the rationale for the choice of cases and controls	17-19
		(*b*) For matched studies, give matching criteria and the number of controls per case	
Variables	7	Clearly define all outcomes, exposures, predictors, potential confounders, and effect modifiers. Give diagnostic criteria, if applicable	17-19
Data sources/ measurement	8*	For each variable of interest, give sources of data and details of methods of assessment (measurement). Describe comparability of assessment methods if there is more than one group	16-19
Bias	9	Describe any efforts to address potential sources of bias	18
Study size	10	Explain how the study size was arrived at	16
Quantitative variables	11	Explain how quantitative variables were handled in the analyses. If applicable, describe which groupings were chosen and why	17-18
Statistical methods	12	(*a*) Describe all statistical methods, including those used to control for confounding	29-30
		(*b*) Describe any methods used to examine subgroups and interactions	29-30
		(*c*) Explain how missing data were addressed	
		(*d*) If applicable, explain how matching of cases and controls was addressed	
		(*e*) Describe any sensitivity analyses	

Results

Participants	13*	(a) Report numbers of individuals at each stage of study—eg numbers potentially eligible, examined for eligibility, confirmed eligible, included in the study, completing follow-up, and analysed	5
		(b) Give reasons for non-participation at each stage	
		(c) Consider use of a flow diagram	
Descriptive data	14*	(a) Give characteristics of study participants (eg demographic, clinical, social) and information on exposures and potential confounders	5
		(b) Indicate number of participants with missing data for each variable of interest	
Outcome data	15*	Report numbers in each exposure category, or summary measures of exposure	5
Main results	16	(*a*) Give unadjusted estimates and, if applicable, confounder-adjusted estimates and their precision (eg, 95% confidence interval). Make clear which confounders were adjusted for and why they were included	5
		(*b*) Report category boundaries when continuous variables were categorized	
		(*c*) If relevant, consider translating estimates of relative risk into absolute risk for a meaningful time period	
Other analyses	17	Report other analyses done—eg analyses of subgroups and interactions, and sensitivity analyses	

Discussion

Key results	18	Summarise key results with reference to study objectives	13
Limitations	19	Discuss limitations of the study, taking into account sources of potential bias or imprecision. Discuss both direction and magnitude of any potential bias	14
Interpretation	20	Give a cautious overall interpretation of results considering objectives, limitations, multiplicity of analyses, results from similar studies, and other relevant evidence	13-15
Generalisability	21	Discuss the generalisability (external validity) of the study results	15

Other information

Funding	22	Give the source of funding and the role of the funders for the present study and, if applicable, for the original study on which the present article is based	30

*Give information separately for cases and controls.

Note: An Explanation and Elaboration article discusses each checklist item and gives methodological background and published examples of transparent reporting. The STROBE checklist is best used in conjunction with this article (freely available on the Web sites of PLoS Medicine at

http://www.plosmedicine.org/, Annals of Internal Medicine at http://www.annals.org/, and Epidemiology at http://www.epidem.com/). Information on the STROBE Initiative is available at http://www.strobe-statement.org.
