# Supplementary material for: Low chorionic villous succinate accumulation associates with recurrent spontaneous abortion risk
Source: Nat Commun. 2021 Jun 8;12:3428. doi: 10.1038/s41467-021-23827-0 (PMC8187647; doi:10.1038/s41467-021-23827-0)
Supplement: Supplementary file 3 — Reporting Summary New [file 41467_2021_23827_MOESM3_ESM.pdf]

nature research

Corresponding author(s): Jian-Yuan Zhao

Last updated by author(s): Jan 28, 2021

Reporting Summary

Nature Research wishes to improve the reproducibility of the work that we publish. This form provides structure for consistency and transparency in reporting. For further information on Nature Research policies, see our Editorial Policies and the Editorial Policy Checklist.

Statistics

For all statistical analyses, confirm that the following items are present in the figure legend, table legend, main text, or Methods section.

n/a	Confirmed	
☐	☒	The exact sample size (*n*) for each experimental group/condition, given as a discrete number and unit of measurement
☐	☒	A statement on whether measurements were taken from distinct samples or whether the same sample was measured repeatedly
☐	☒	The statistical test(s) used AND whether they are one- or two-sided<br>*Only common tests should be described solely by name; describe more complex techniques in the Methods section.*
☒	☐	A description of all covariates tested
☐	☒	A description of any assumptions or corrections, such as tests of normality and adjustment for multiple comparisons
☐	☒	A full description of the statistical parameters including central tendency (e.g. means) or other basic estimates (e.g. regression coefficient) AND variation (e.g. standard deviation) or associated estimates of uncertainty (e.g. confidence intervals)
☐	☒	For null hypothesis testing, the test statistic (e.g. *F*, *t*, *r*) with confidence intervals, effect sizes, degrees of freedom and *P* value noted<br>*Give P values as exact values whenever suitable.*
☒	☐	For Bayesian analysis, information on the choice of priors and Markov chain Monte Carlo settings
☒	☐	For hierarchical and complex designs, identification of the appropriate level for tests and full reporting of outcomes
☒	☐	Estimates of effect sizes (e.g. Cohen's *d*, Pearson's *r*), indicating how they were calculated

Our web collection on statistics for biologists contains articles on many of the points above.

Software and code

Policy information about availability of computer code

Data collection	No software was used for data collection.
Data analysis	Prism version 6.0, TOPSPIN version 3.6.0, AMIX software package version 3.8.3, Image-Quanta software version 1.0 and R version 2.17

For manuscripts utilizing custom algorithms or software that are central to the research but not yet described in published literature, software must be made available to editors and reviewers. We strongly encourage code deposition in a community repository (e.g. GitHub). See the Nature Research guidelines for submitting code & software for further information.

Data

Policy information about availability of data

All manuscripts must include a data availability statement. This statement should provide the following information, where applicable:

- Accession codes, unique identifiers, or web links for publicly available datasets
- A list of figures that have associated raw data
- A description of any restrictions on data availability

The dataset of patients hospitalized at Shanghai First Maternity and Infant Hospital are stored on a server at Shanghai First Maternity and Infant Hospital. All data generated and/or analyzed in the current study are available from the corresponding author on reasonable request.

Field-specific reporting

Life sciences study design

All studies must disclose on these points even when the disclosure is negative.

Sample size	To estimate the proper sample size for our study, we presumed the concentration of target metabolite fall into the range of 0.4-0.6 µmol/g and performed a two-sample T-test power analysis. When the threshold value of power is 0.9, the minimal sufficient sample size to tell the significant difference of metabolite concentration between the case group and the control group is n=12. When the threshold value of power is increased to 0.95, the minimal sufficient sample size is n=15. So in our study design, we extended the sample size to n=30 RSA patients and n=30 healthy controls to fully meet statistical requirements.
Data exclusions	Among these patients, we excluded cases with co-existing health problems, including 1) infection, by taking body temperature and blood examination, 2) endocrine or metabolic diseases, including polycystic ovarian syndrome, hyperprolactinemia, hypothyroidism, hyperthyroidism, diabetes mellitus, etc., by testing hormone levels, thyroid function, and blood glucose levels, 3) chromosomal abnormalities, by conducting villi low-density chip to exclude chromosome number abnormalities, 4) anatomical abnormalities, including congenital malformation of uterus, cervical incompetence, intrauterine adhesions, uterine fibroids, adenomyosis, etc., 5) autoimmune diseases, including anti-phospholipid antibody syndrome, systemic lupus erythematosus, Sjogren's syndrome, etc., by testing immune index. The above criteria were designed to exclude known causes or risk factors of RSA.
Replication	We have 3 replications for each experiment. The results are repeatable.
Randomization	Human samples were not randomized. For animal study, all the animals were randomly grouped for experiments.
Blinding	For experiments using cell lines the investigators were not blinded during data acquisition and analysis. The application of treatments and processing procedures negated the possibility of blinding but there was no human bias given all data was collected independently using instrumentation. Similarly, in the animal experiments the investigators were not blinded to the group allocation. Two observers measured volumes/weights to alleviate human bias in these data. For experiments involving human tissues, sample IDs were coded and the investigator was not aware of the group allocation during data acquisition. Group allocations were decoded afterwards for the purpose of data analysis.

Behavioural & social sciences study design

All studies must disclose on these points even when the disclosure is negative.

Study description	*Briefly describe the study type including whether data are quantitative, qualitative, or mixed-methods (e.g. qualitative cross-sectional, quantitative experimental, mixed-methods case study).*
Research sample	*State the research sample (e.g. Harvard university undergraduates, villagers in rural India) and provide relevant demographic information (e.g. age, sex) and indicate whether the sample is representative. Provide a rationale for the study sample chosen. For studies involving existing datasets, please describe the dataset and source.*
Sampling strategy	*Describe the sampling procedure (e.g. random, snowball, stratified, convenience). Describe the statistical methods that were used to predetermine sample size OR if no sample-size calculation was performed, describe how sample sizes were chosen and provide a rationale for why these sample sizes are sufficient. For qualitative data, please indicate whether data saturation was considered, and what criteria were used to decide that no further sampling was needed.*
Data collection	*Provide details about the data collection procedure, including the instruments or devices used to record the data (e.g. pen and paper, computer, eye tracker, video or audio equipment) whether anyone was present besides the participant(s) and the researcher, and whether the researcher was blind to experimental condition and/or the study hypothesis during data collection.*
Timing	*Indicate the start and stop dates of data collection. If there is a gap between collection periods, state the dates for each sample cohort.*
Data exclusions	*If no data were excluded from the analyses, state so OR if data were excluded, provide the exact number of exclusions and the rationale behind them, indicating whether exclusion criteria were pre-established.*
Non-participation	*State how many participants dropped out/declined participation and the reason(s) given OR provide response rate OR state that no participants dropped out/declined participation.*
Randomization	*If participants were not allocated into experimental groups, state so OR describe how participants were allocated to groups, and if allocation was not random, describe how covariates were controlled.*

Ecological, evolutionary & environmental sciences study design

All studies must disclose on these points even when the disclosure is negative.

Study description	*Briefly describe the study. For quantitative data include treatment factors and interactions, design structure (e.g. factorial, nested, hierarchical), nature and number of experimental units and replicates.*

Research sample	*Describe the research sample (e.g. a group of tagged Passer domesticus, all Stenocereus thurberi within Organ Pipe Cactus National Monument), and provide a rationale for the sample choice. When relevant, describe the organism taxa, source, sex, age range and any manipulations. State what population the sample is meant to represent when applicable. For studies involving existing datasets, describe the data and its source.*
Sampling strategy	*Note the sampling procedure. Describe the statistical methods that were used to predetermine sample size OR if no sample-size calculation was performed, describe how sample sizes were chosen and provide a rationale for why these sample sizes are sufficient.*
Data collection	*Describe the data collection procedure, including who recorded the data and how.*
Timing and spatial scale	*Indicate the start and stop dates of data collection, noting the frequency and periodicity of sampling and providing a rationale for these choices. If there is a gap between collection periods, state the dates for each sample cohort. Specify the spatial scale from which the data are taken*
Data exclusions	*If no data were excluded from the analyses, state so OR if data were excluded, describe the exclusions and the rationale behind them, indicating whether exclusion criteria were pre-established.*
Reproducibility	*Describe the measures taken to verify the reproducibility of experimental findings. For each experiment, note whether any attempts to repeat the experiment failed OR state that all attempts to repeat the experiment were successful.*
Randomization	*Describe how samples/organisms/participants were allocated into groups. If allocation was not random, describe how covariates were controlled. If this is not relevant to your study, explain why.*
Blinding	*Describe the extent of blinding used during data acquisition and analysis. If blinding was not possible, describe why OR explain why blinding was not relevant to your study.*

Did the study involve field work? ☐ Yes ☐ No

Field work, collection and transport

Field conditions	*Describe the study conditions for field work, providing relevant parameters (e.g. temperature, rainfall).*
Location	*State the location of the sampling or experiment, providing relevant parameters (e.g. latitude and longitude, elevation, water depth).*
Access & import/export	*Describe the efforts you have made to access habitats and to collect and import/export your samples in a responsible manner and in compliance with local, national and international laws, noting any permits that were obtained (give the name of the issuing authority, the date of issue, and any identifying information).*
Disturbance	*Describe any disturbance caused by the study and how it was minimized.*

Reporting for specific materials, systems and methods

We require information from authors about some types of materials, experimental systems and methods used in many studies. Here, indicate whether each material, system or method listed is relevant to your study. If you are not sure if a list item applies to your research, read the appropriate section before selecting a response.

Materials & experimental systems

n/a	Involved in the study	
☐	☒	Antibodies
☐	☒	Eukaryotic cell lines
☒	☐	Palaeontology and archaeology
☐	☒	Animals and other organisms
☐	☒	Human research participants
☒	☐	Clinical data
☒	☐	Dual use research of concern

Methods

n/a	Involved in the study	
☒	☐	ChIP-seq
☒	☐	Flow cytometry
☒	☐	MRI-based neuroimaging

Antibodies

Antibodies used

anti-HK1 (#2024;CST; 1:1000 dilution)
anti-GPI (#57893S; CST; 1:1000 dilution)
anti-TPI (10713-1-AP; Proteintech; 1:500 dilution)
anti-PGK1 (17811-1-AP; Proteintech; 1:2000 dilution)
anti-ENO1 (11204-1-AP; Proteintech; 1:500 dilution)
anti-PKM2 (# 4053; CST; 1:2000 dilution)
anti-6PGD (#13389S; CST; 1:1500 dilution)
anti-TKT (11039-1-AP; Proteintech; 1:2000 dilution)
anti-PDHA1 (#3205; CST; 1:2000 dilution)

anti-CS (#14309;CST; 1:500 dilution)
anti-IDH1 (#66969; CST; 1:1000 dilution)
anti-IDH2 (#56439; CST; 1:1000 dilution)
anti-IDH3A (15909-1-AP; Proteintech; 1:1000 dilution)
anti-OGDH (# 26865; CST; 1:1000 dilution)
anti-SUCLA2 (12627-1-AP; Proteintech; 1:5000 dilution)
anti-SDHA (14865-1-AP; Proteintech; 1:2000 dilution)
anti-SDHB (10620-1-AP; Proteintech; 1:1000 dilution)
anti-SDHC (14575-1-AP; Proteintech; 1:1000 dilution)
anti-FH (#4567; CST; 1:1000 dilution)
anti-MDH2 (15462-1-AP; Proteintech; 1:1000 dilution)
anti-MBD1 (ab108510; Abcam; 1:1000 dilution)
anti-MBD2 (55200-1-AP; Proteintech; 1:1000 dilution)
anti-MBD3 (14258-1-AP; Proteintech; 1:1000 dilution)
anti-MBD4 (11270-1-AP; Proteintech; 1:1000 dilution)
anti-MBD5 (15961-1-AP; Proteintech; 1:1000 dilution)
anti-MBD6 (ab204403; Abcam; 1:1000 dilution)
anti-MECP2 (10861-1-AP; Proteintech; 1:1000 dilution)
anti-5-hmC (39769; ActiveMotif; 1:1000 dilution)
anti-HIF1a (# 36169; CST; 1:500 dilution)
anti-IL-1β (#12703; CST; 1:1000 dilution)
anti-CXCR4 (11073-2-AP; Proteintech; 1:500 dilution)
anti-VHL (#68547S;CST; 1:1000 dilution)
anti-PHD2 (#4835; CST; 1:1000 dilution)
HRP-conjugated anti-rabbit IgG secondary antibody (A00098; Genscript; 1:3000 dilution)

Validation

anti-HK1(citation:Xanthohumol inhibits colorectal cancer cells via downregulation of Hexokinases II-mediated glycolysis.)
anti-GPI(manufacture’s website: https://www.cst-c.com.cn/products/primary-antibodies/gpi-antibody/57893?site-searchtype=Products&N=4294956287&Ntt=57893&fromPage=plp&_requestid=825307)
anti-TPI(citation:The Long Noncoding RNA Lncenc1 Maintains Naive States of Mouse ESCs by Promoting the Glycolysis Pathway.)
anti-PGK1 (citation:Long non-coding RNA GBCDRlnc1 induces chemoresistance of gallbladder cancer cells by activating autophagy.)
anti-ENO1(citation: Enolase 1 stimulates glycolysis to promote chemoresistance in gastric cancer.)
anti-PKM2(citation: The M2 Splice Isoform of Pyruvate Kinase Is Important for Cancer Metabolism and Tumour Growth.)
anti-6PGD(citation: 4-hydroxyphenylpyruvate dioxygenase promotes lung cancer growth via pentose phosphate pathway (PPP) flux mediated by LKB1-AMPK/HDAC10/G6PD axis.)
anti-TKT(citation: Malignant pleural effusion cells show aberrant glucose metabolism gene expression.)
anti-PDHA1(citation:O-GlcNAcylation of PGK1 coordinates glycolysis and TCA cycle to promote tumor growth.)
anti-CS(citation:Hypoxia-reprogrammed tricarboxylic acid cycle promotes the growth of human breast tumorigenic cells.)
anti-IDH1 (manufacture’s website: https://www.cst-c.com.cn/products/primary-antibodies/gpi-antibody/57893?site-searchtype=Products&N=4294956287&Ntt=57893&fromPage=plp&_requestid=825307)
anti-IDH2 (citation:Lysine 68 acetylation directs MnSOD as a tetrameric detoxification complex versus a monomeric tumor promoter.)
anti-IDH3A(citation: Mouse Idh3a mutations cause retinal degeneration and reduced mitochondrial function.)
anti-OGDH (manufacture’s website: https://www.cst-c.com.cn/products/primary-antibodies/gpi-antibody/57893?site-searchtype=Products&N=4294956287&Ntt=57893&fromPage=plp&_requestid=825307)
anti-SUCLA2(citation:Two transgenic mouse models for beta subunit components of succinate-CoA ligase yielding pleiotropic metabolic alterations.)
anti-SDHA(citation:Lin28/let-7 axis regulates aerobic glycolysis and cancer progression via PDK1.)
anti-SDHB(citation:SDHB deficiency promotes TGF"-mediated invasion and metastasis of colorectal cancer through transcriptional repression complex SNAIL1-SMAD3/4.)
anti-SDHC(citation:Quantitative proteomics in A30P*A53T !-synuclein transgenic mice reveals upregulation of Sel1l.)
anti-FH(citation:Fumarate hydratase inactivation in hereditary leiomyomatosis and renal cell cancer is synthetic lethal with ferroptosis induction.)
anti-MDH2(citation: Human METTL12 is a mitochondrial methyltransferase that modifies citrate synthase.)
anti-HIF1a(citation:Ginkgo biloba Extract Reduces Hippocampus Inflammatory Responses, Improves Cardiac Functions And Depressive Behaviors In A Heart Failure Mouse Model.)
anti-IL-1β (citation:Low-Dose Mitomycin C Decreases the Postoperative Recurrence Rate of Pterygium by Perturbing NLRP3 Inflammatory Signalling Pathway and Suppressing the Expression of Inflammatory Factors.)
anti-CXCR4 (citation:Electroacupuncture at ST36 Increases Bone Marrow-Derived Interstitial Cells of Cajal via the SDF-1/CXCR4 and mSCF/Kit-ETV1 Pathways in the Stomach of Diabetic Mice.)
anti-VHL (citation:Targeting bromodomain-containing protein 4 (BRD4) inhibits MYC expression in colorectal cancer cells.)
anti-PHD2 (citation:UCP2-induced hypoxia promotes lipid accumulation and tubulointerstitial fibrosis during ischemic kidney injury.)
anti-MBD1 (citation:Epigenetic biomarker screening by FLIM-FRET for combination therapy in ER+ breast cancer.)
anti-MBD2 (citation:The Expression of MBD6 Is Associated with Tumor Size in Uterine Leiomyomas.)
anti-MBD3 (citation:Compensatory functions of histone deacetylase 1 (HDAC1) and HDAC2 regulate transcription and apoptosis during mouse oocyte development.)
anti-MBD4 (citation:The Expression of MBD6 Is Associated with Tumor Size in Uterine Leiomyomas.)
anti-MBD5 (citation:Distinct Pathogenic Genes Causing Intellectual Disability and Autism Exhibit a Common Neuronal Network Hyperactivity Phenotype.)
anti-MBD6 (manufacture’s website: https://www.abcam.cn/mbd6-antibody-ab204403.html?productWallTab=Abreviews)

anti-MECP2 (citation:Specificity of Antinuclear Autoantibodies Recognizing the Dense Fine Speckled Nuclear Pattern: Preferential Targeting of DFS70/LEDGFp75 Over its Interacting Partner MeCP2.)
anti-5-hmc (citation:A genome-scale map of DNA methylation turnover identifies site-specific dependencies of DNMT and TET activity.)
HRP-conjugated anti-rabbit IgG secondary antibody(citation:Sestrin2 as Serum Protein Marker and Potential Therapeutic Target for Parkinson's Disease)

Eukaryotic cell lines

Policy information about cell lines

Cell line source(s)	HEK293T cells (ATCC Number: CRL-3216), JEG-3 cells (ATCC Number: HTB-36) and HTR-8 cells (ATCC Number: CRL-3271)were used in this study. All cell lines were obtained from the American Type Culture Collection (ATCC).
Authentication	All the cell lines used were authenticated using Short Tandem Repeat (STR) analysis.
Mycoplasma contamination	All cell lines tested negative for mycoplasma contamination.
Commonly misidentified lines (See ICLAC register)	This study did not involve commonly misidentified cell lines.

Palaeontology and Archaeology

Specimen provenance	*Provide provenance information for specimens and describe permits that were obtained for the work (including the name of the issuing authority, the date of issue, and any identifying information).*
Specimen deposition	*Indicate where the specimens have been deposited to permit free access by other researchers.*
Dating methods	*If new dates are provided, describe how they were obtained (e.g. collection, storage, sample pretreatment and measurement), where they were obtained (i.e. lab name), the calibration program and the protocol for quality assurance OR state that no new dates are provided.*

☐ Tick this box to confirm that the raw and calibrated dates are available in the paper or in Supplementary Information.

Ethics oversight	*Identify the organization(s) that approved or provided guidance on the study protocol, OR state that no ethical approval or guidance was required and explain why not.*

Note that full information on the approval of the study protocol must also be provided in the manuscript.

Animals and other organisms

Policy information about studies involving animals; ARRIVE guidelines recommended for reporting animal research

Laboratory animals	For the low succinate mouse model, eight-week-old female C57BL/6 mice (weight: 20–23 g; n = 24) were divided into three equal groups using a random number table by body weight, age, and family.<br>For the recurrent abortion mouse model,eight-week-old CBA/J female mice were mated to eight-week-old DBA/2 male mice. As the control, eight-week-old CBA/J female mice were mated to eight-week-old BALB/C male mice as control mating combination.<br>Mice were housed in the animal care facility of Anhui Medical University under standard pathogen-free conditions with a 12 h light/dark schedule and provided with food and water ad libitum, temperature was between 20 and 24 °C and relative humidity between 45 and 65 rH.
Wild animals	The study did not involve wild animals.
Field-collected samples	The study did not involve samples collected from the field.
Ethics oversight	The studies with research animals complied with the Experimental Animal Management Regulations of China. All experiments were reviewed and approved by the Institutional Animal Care and Use Committee of the institute at Anhui Medical University.

Note that full information on the approval of the study protocol must also be provided in the manuscript.

Human research participants

Policy information about studies involving human research participants

Population characteristics	First-trimester villous and decidual tissues were obtained from the placentas of individuals with clinically normal pregnancies (age ranged from 22 to 34, 28 ± 5 years; gestational age at sampling, 52 ± 9 days, mean ± SD) that had been terminated for non-medical reasons. Second-trimester placentas were obtained from individuals with clinically normal pregnancies (age ranged from 22 to 34, 28 ± 6 years) that had been terminated due to unplanned pregnancy or family planning. Thirdtrimester placentas were obtained from individuals (age ranged from 22 to 34, 28 ± 5 years) who had undergone normal vaginal delivery. Villous samples were also obtained from patients with RSA (age ranged from 22 to 34, 28 ± 6 years, gestational age at abortion, 53 ± 7 days) who had experienced more than three unexplained and consecutive spontaneous abortions at a gestational age matched to that of the normal control group.

Recruitment	All patients with normal pregnancy and patients with RSA who underwent induced abortion in Shanghai First Maternity and Infant Hospital provided written informed consent. All clinical samples were collected betweenJune 1st 2016 and Aug 31th 2020 at Shanghai First Maternity and Infant Hospital. Among these patients, a series of criteriawere designed to exclude known causes or risk factors of RSA such as infection, endocrine or metabolic diseases, chromosomal abnormalities, anatomical abnormalities and autoimmune diseases. However, there still might be some unknown confounding factor which may lead to unaddressed confounding bias.
Ethics oversight	Study design and conduct were approved and supervised by the Ethics Committee of Shanghai First Maternity and Infant Hospital (https://www.51mch.com/news/content/id/287/pid/18552) through Ethics Vote KS18133 in accordance to the criteria set by the Declaration of Helsinki. The Ethics Committee of Shanghai First Maternity and Infant Hospital can be contacted by writing to the Office of Ethics Committee, Shanghai First Maternity and Infant Hospital, 2699 West Gaoke Road, Pudong District, 201300 Shanghai or sending an email to shsdyfybjyyxllwyh@126.com or by calling +86-021-20261211.

Note that full information on the approval of the study protocol must also be provided in the manuscript.

Clinical data

Policy information about clinical studies

All manuscripts should comply with the ICMJEguidelines for publication of clinical research and a completedCONSORT checklist must be included with all submissions.

Clinical trial registration	*Provide the trial registration number from ClinicalTrials.gov or an equivalent agency.*
Study protocol	*Note where the full trial protocol can be accessed OR if not available, explain why.*
Data collection	*Describe the settings and locales of data collection, noting the time periods of recruitment and data collection.*
Outcomes	*Describe how you pre-defined primary and secondary outcome measures and how you assessed these measures.*

Dual use research of concern

Policy information about dual use research of concern

Hazards

Could the accidental, deliberate or reckless misuse of agents or technologies generated in the work, or the application of information presented in the manuscript, pose a threat to:

No	Yes	
☐	☐	Public health
☐	☐	National security
☐	☐	Crops and/or livestock
☐	☐	Ecosystems
☐	☐	Any other significant area

Experiments of concern

Does the work involve any of these experiments of concern:

No	Yes	
☐	☐	Demonstrate how to render a vaccine ineffective
☐	☐	Confer resistance to therapeutically useful antibiotics or antiviral agents
☐	☐	Enhance the virulence of a pathogen or render a nonpathogen virulent
☐	☐	Increase transmissibility of a pathogen
☐	☐	Alter the host range of a pathogen
☐	☐	Enable evasion of diagnostic/detection modalities
☐	☐	Enable the weaponization of a biological agent or toxin
☐	☐	Any other potentially harmful combination of experiments and agents

ChIP-seq

Data deposition

☐ Confirm that both raw and final processed data have been deposited in a public database such as GEO.

☐ Confirm that you have deposited or provided access to graph files (e.g. BED files) for the called peaks.

Data access links
May remain private before publication.

For "Initial submission" or "Revised version" documents, provide reviewer access links. For your "Final submission" document, provide a link to the deposited data.

Files in database submission

Provide a list of all files available in the database submission.

Genome browser session
(e.g. UCSC)

Provide a link to an anonymized genome browser session for "Initial submission" and "Revised version" documents only, to enable peer review. Write "no longer applicable" for "Final submission" documents.

Methodology

Replicates

Describe the experimental replicates, specifying number, type and replicate agreement.

Sequencing depth

Describe the sequencing depth for each experiment, providing the total number of reads, uniquely mapped reads, length of reads and whether they were paired- or single-end.

Antibodies

Describe the antibodies used for the ChIP-seq experiments; as applicable, provide supplier name, catalog number, clone name, and lot number.

Peak calling parameters

Specify the command line program and parameters used for read mapping and peak calling, including the ChIP, control and index files used.

Data quality

Describe the methods used to ensure data quality in full detail, including how many peaks are at FDR 5% and above 5-fold enrichment.

Software

Describe the software used to collect and analyze the ChIP-seq data. For custom code that has been deposited into a community repository, provide accession details.

Flow Cytometry

Plots

Confirm that:

- [ ] The axis labels state the marker and fluorochrome used (e.g. CD4-FITC).
- [ ] The axis scales are clearly visible. Include numbers along axes only for bottom left plot of group (a 'group' is an analysis of identical markers).
- [ ] All plots are contour plots with outliers or pseudocolor plots.
- [ ] A numerical value for number of cells or percentage (with statistics) is provided.

Methodology

Sample preparation

Describe the sample preparation, detailing the biological source of the cells and any tissue processing steps used.

Instrument

Identify the instrument used for data collection, specifying make and model number.

Software

Describe the software used to collect and analyze the flow cytometry data. For custom code that has been deposited into a community repository, provide accession details.

Cell population abundance

Describe the abundance of the relevant cell populations within post-sort fractions, providing details on the purity of the samples and how it was determined.

Gating strategy

Describe the gating strategy used for all relevant experiments, specifying the preliminary FSC/SSC gates of the starting cell population, indicating where boundaries between "positive" and "negative" staining cell populations are defined.

- [ ] Tick this box to confirm that a figure exemplifying the gating strategy is provided in the Supplementary Information.

Magnetic resonance imaging

Experimental design

Design type

Indicate task or resting state; event-related or block design.

Design specifications

Specify the number of blocks, trials or experimental units per session and/or subject, and specify the length of each trial or block (if trials are blocked) and interval between trials.

Behavioral performance measures

State number and/or type of variables recorded (e.g. correct button press, response time) and what statistics were used to establish that the subjects were performing the task as expected (e.g. mean, range, and/or standard deviation across subjects).

Acquisition

Imaging type(s) | *Specify: functional, structural, diffusion, perfusion.*

Field strength | *Specify in Tesla*

Sequence & imaging parameters | *Specify the pulse sequence type (gradient echo, spin echo, etc.), imaging type (EPI, spiral, etc.), field of view, matrix size, slice thickness, orientation and TE/TR/flip angle.*

Area of acquisition | *State whether a whole brain scan was used OR define the area of acquisition, describing how the region was determined.*

Diffusion MRI ☐ Used ☐ Not used

Preprocessing

Preprocessing software | *Provide detail on software version and revision number and on specific parameters (model/functions, brain extraction, segmentation, smoothing kernel size, etc.).*

Normalization | *If data were normalized/standardized, describe the approach(es): specify linear or non-linear and define image types used for transformation OR indicate that data were not normalized and explain rationale for lack of normalization.*

Normalization template | *Describe the template used for normalization/transformation, specifying subject space or group standardized space (e.g. original Talairach, MNI305, ICBM152) OR indicate that the data were not normalized.*

Noise and artifact removal | *Describe your procedure(s) for artifact and structured noise removal, specifying motion parameters, tissue signals and physiological signals (heart rate, respiration).*

Volume censoring | *Define your software and/or method and criteria for volume censoring, and state the extent of such censoring.*

Statistical modeling & inference

Model type and settings | *Specify type (mass univariate, multivariate, RSA, predictive, etc.) and describe essential details of the model at the first and second levels (e.g. fixed, random or mixed effects; drift or auto-correlation).*

Effect(s) tested | *Define precise effect in terms of the task or stimulus conditions instead of psychological concepts and indicate whether ANOVA or factorial designs were used.*

Specify type of analysis: ☐ Whole brain ☐ ROI-based ☐ Both

Statistic type for inference (See Eklund et al. 2016) | *Specify voxel-wise or cluster-wise and report all relevant parameters for cluster-wise methods.*

Correction | *Describe the type of correction and how it is obtained for multiple comparisons (e.g. FWE, FDR, permutation or Monte Carlo).*

Models & analysis

n/a	Involved in the study
☐	☐ Functional and/or effective connectivity
☐	☐ Graph analysis
☐	☐ Multivariate modeling or predictive analysis

Functional and/or effective connectivity | *Report the measures of dependence used and the model details (e.g. Pearson correlation, partial correlation, mutual information).*

Graph analysis | *Report the dependent variable and connectivity measure, specifying weighted graph or binarized graph, subject- or group-level, and the global and/or node summaries used (e.g. clustering coefficient, efficiency, etc.).*

Multivariate modeling and predictive analysis | *Specify independent variables, features extraction and dimension reduction, model, training and evaluation metrics.*
